# Prevalence, Pattern, and Reasons for Self-Medication: A Community-Based Cross-Sectional Study From Central India

**DOI:** 10.7759/cureus.33917

**Published:** 2023-01-18

**Authors:** Pragati Rathod, Sarita Sharma, Ujwala Ukey, Bhagyashri Sonpimpale, Suresh Ughade, Uday Narlawar, Sanju Gaikwad, Parvati Nair, Pushkar Masram, Snigdha Pandey

**Affiliations:** 1 Community Medicine, Government Medical College (GMC), Nagpur, IND

**Keywords:** self-medication, community survey, cross sectional studies, reasons, pattern

## Abstract

Introduction

Self-medication is an important public health problem, with varied prevalence across the world. The high prevalence of self-medication in India is one of the important factors contributing to the development of antimicrobial resistance. Self-medication without medical guidance can lead to inappropriate, incorrect, or undue therapy, missed diagnosis, delays in appropriate treatment, pathogen resistance, and increased morbidity. The growing trend of self-medication can be attributed to various factors like the urge for self-care, sympathy toward sick family members, inaccessible health services and nonavailability of drugs, time and financial constraints, ignorance, misbeliefs, extensive advertisement and availability of drugs in places other than drug shops.

Methodology

The present community-based descriptive cross-sectional study was conducted in an urban field practice area of a tertiary health care center (UHTC) in Central India. Individuals above 18 years of age and present at home at the time of the house-to-house survey comprised the study participants. A total of 400 participants were enrolled in the study. Data were collected using a predesigned and pretested questionnaire by the face-to-face interview technique.

Results

The prevalence of self-medication in the area was 60 % (240). The most widely used drugs for self-medication were analgesics (159; 66.25%) and antipyretics (142; 59.16%). Common ailments for which self-medication was used frequently were fever, body aches, common cold, and cough. It was observed that female participants were twice more likely to self-medicate as compared to male participants (Odds Ratio (OR): 2.04; Prevalence (p) = 0.014, Confidence Interval (CI) 95% = 1.15-3.62). Additionally, those having education above the high school level had more chances of self-medicating than those educated less than high school (OR: 1.25; p≤0.014, CI 95%=1.05-1.50). The commonest reasons for resorting to self-medication as per the findings of the present study are that it saves time and the condition was not serious enough to warrant a physician’s consultation.

## Introduction

Practiced globally, self-medication is an important public health problem, with prevalence ranging from 11.7% to 92% across the world [1-6]. World Health Organization (WHO) has defined self-medication as the use of drugs to treat self-diagnosed disorders or symptoms or, the intermittent or continued use of prescribed drugs for chronic or recurrent disease or symptoms. In practice, it also includes the use of the medication of family members, especially where the treatment of children or the elderly is involved [7]. The practice of self-medication is widely prevalent in developing countries like India where there is relatively easy access to a wide range of over-the-counter (OTC) drugs with proven efficiency and safety [8]. The high prevalence of self-medication in India is one of the important factors attributing to the development of antimicrobial resistance. Furthermore, it can also cause serious health hazards, leading to prolonged suffering [9].

Self-medication without sufficient knowledge may lead to the irrational use of drugs causing serious negative health impacts and increased economic burden. Rampant irrational use of drugs without medical guidance may result in a greater probability of inappropriate, incorrect, or undue therapy, missed diagnosis, delays in appropriate treatment, pathogen resistance, and increased morbidity [10].

On the other hand, if practiced responsibly and appropriately, self‐medication can readily relieve acute medical problems, save the time spent waiting to see a doctor as well as the traveling time, may be economical, and can be life-saving in emergency medical situations. Some governments are increasingly encouraging self-care for minor illnesses, including self-medication [10]. It is now a well-accepted fact that self‐care in the form of responsible self‐medication can be beneficial for patients, healthcare providers, the pharmaceutical industry, and governments. However, appropriate health information is of utmost importance for self‐medicating. Thus self-medication can be described as a double-edged sword for its users having both beneficial and harmful effects. Numerous factors are thought to contribute to self-medication, and this can vary in a region-specific manner with a few of them being lifestyle, easy drug access, socioeconomic factors, etc. [11]. The growing trend of self-medication can be attributed to various factors like the urge for self-care, sympathy toward sick family members, inaccessible health services and nonavailability of drugs, and time and financial constraints. Ignorance, misbeliefs, extensive advertisement, and availability of drugs in shops other than drug stores also contribute to this increasing trend [12]. With this background, the present cross-sectional community-based study was conducted to find out the prevalence, pattern, and reasons for self-medication.

## Materials and methods

Study design, setting, and duration

The present community-based descriptive cross-sectional study was conducted in an urban field practice area of a tertiary health care center in Central India. The data was collected over seven months, from June 2019 to December 2019.

Study population

Individuals above 18 years of age and present at home at the time of the house-to-house survey comprised the study participants. One individual per household was included in the study and in case there was more than one individual above 18 years of age in the household at the time of the survey, the eldest one was interviewed. The eldest available adult (>18 years) in the household who was willing to participate in the study was included. Doctors, pharmacists, and other healthcare workers with knowledge of medications were excluded.

Sample size calculation

Considering the prevalence of self-medication practices to be 51.5% as per the study conducted by Limaye D et al. [2], the minimum estimated sample size was calculated to be 383 using the formula 4p (1−p)/d^2^, where p is prevalence and d is precision with a desired confidence limit of 95% and relative precision of 5%. However, the actual sample taken was 400.

Sampling technique

A simple random sampling was conducted. On entering the field practice area of the urban health training center (UHTC), a pen was rotated at the entry point to decide the direction for moving in the study area. Then, a coin was tossed to choose the side of the street (right or left ). The last two digits of a randomly selected currency note were then considered to decide the number of footsteps to select the first household randomly. The same procedure was applied on each day of data collection, taking care that the survey was not carried out in the same area again. Thus, a representative sample from the study group was collected.

Data collection

Data were collected using the face-to-face interview technique. The data collection tool was a predesigned, pretested questionnaire having questions in a vernacular language (Hindi or Marathi). The questionnaire was constructed by the lead researcher and was reviewed by an expert panel for content validity and reliability. Questions were derived after an extensive review of the available literature on the topic. It was piloted on 5% of the subjects and necessary modifications were made accordingly. These subjects were excluded from the final analysis. The questionnaire consisted of two sections. Questions seeking information on participants' sociodemographic characteristics, such as age, residence, religion, marital status, education, occupation, type of family, and socioeconomic status, constituted part A. Socioeconomic status was assessed by using Modified B.G. Prasad’s scale. Part B comprised questions dealing with self-medication practices during the previous year, including frequency and patterns of self-medication, types of medicines used, reasons for the same, etc. The operational definition of self-medication taken was the use of over-the-counter drugs or any allopathic drug for self-treatment, without prior consultation with a certified allopathic doctor with a minimum MBBS degree [7].

Ethical considerations

Approval from the Institutional Ethics Committee (IEC) of Government Medical College, Nagpur, was obtained vide letter number 205/EC/Pharmac/GMC/Nagpur. Participation in the study was entirely voluntary, and the study subjects were ensured complete confidentiality and anonymity. Written informed consent from study subjects was obtained after explaining the nature and purpose of the study.

Data management

The final data were summarized into percentages and analyzed by cross-tabulations for various variables. Analysis was done using Epi Info version 7.2.2.5 [13]. In bivariate analysis, chi-square values were calculated wherever appropriate and a p-value less than 0.05 was considered statistically significant. Binary logistic regression was also applied and an odds ratio with a 95% confidence interval was calculated.

## Results

**Figure 1:**
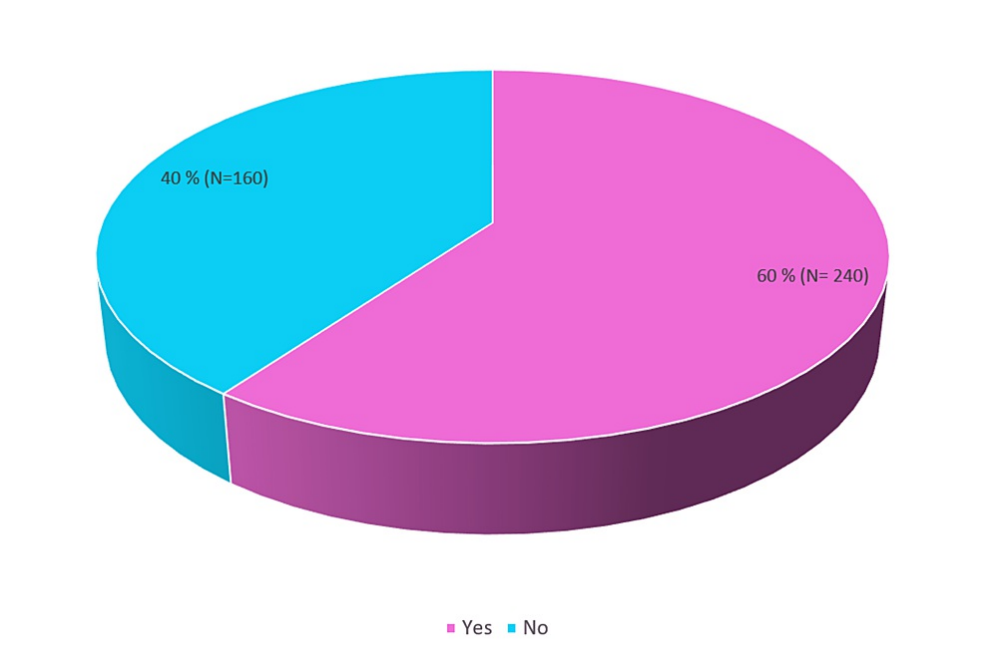
Prevalence of self-medication

A total of 400 households in the urban field practice area were surveyed, and one participant from each household was interviewed. The surveyed population had a mean age of 42 years and a Standard Deviation (SD) of 15.75 and ranged from 18 years to a maximum of 80 years. The prevalence of self-medication in the area was 60% (240) (Figure 1).

From Table 1, it can be seen that self-medication was practiced in all the age groups, but there was no statistically significant difference in self-medication prevalence according to age group, education, socioeconomic status, and type of family. However, the prevalence of self-medication was higher among female participants and those who were unemployed or homemakers, and this difference was found to be statistically significant. On application of binary logistic regression, it was observed that female participants were twice more likely to self-medicate as compared to male participants (Odds Ratio (OR): 2.04; p (prevalence) = 0.014, Confidence Interval (CI) 95% = 1.15-3.62). Additionally, those having an education above the high school level had more chances of self-medicating than those educated less than high school (OR: 1.25; p≤0.014, CI 95% = 1.05-1.50).

**Table 1:** Sociodemographic factors related to self-medication * Significant p-value ** Socioeconomic status was calculated using a modified B.G. Prasad's scale

Variable	Category	All subjects	Practicing self-medication				P-value
			Yes(240)		No (160)		
		Number	Number	Percentage	Number	Percentage	
Age group (years)	18 - 30	110	69	28.75	41	25.63	0.3
	31 - 50	164	91	37.92	73	45.63	
	> 51	126	80	33.33	46	28.75	
Gender	Female	197	133	55.42	64	40	0.003*
	Male	203	107	44.58	96	60	
Education	Below High School	110	73	30.42	37	23.13	0.1
	High School and Above	290	167	69.58	123	76.88	
Occupation	Unemployed and Homemakers	161	112	46.67	49	30.63	0.00135*
	Employed	239	128	53.33	111	69.38	
Type of family	Nuclear	254	146	60.83	108	67.5	0.17
	Joint & Three Generations	146	94	39.17	52	32.5	
Socioeconomic status **	Class I to III	135	77	32.08	58	36.25	0.4
	Class IV to V	256	163	67.92	102	63.75	

**Figure 2:**
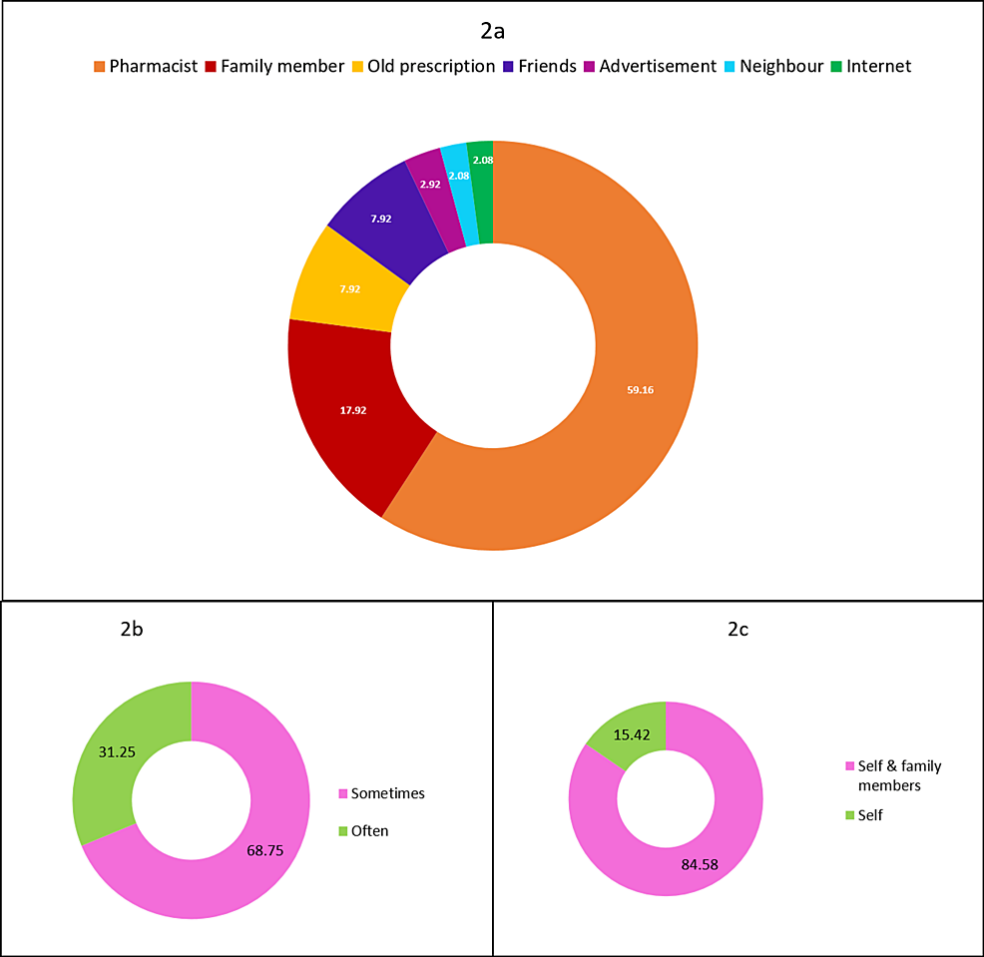
Self-medication: source of information, frequency, and use

Figure 2 shows the sources of information, frequency, and use of self-medication. The main source of information about the drugs for self-medication were local pharmacists for 142 (59.17%) respondents and family members in the case of 43 (17.92%) respondents. Other interesting sources were friends (19; 7.92%), advertisements (7; 2.92%), and the internet (5; 2.08%). Out of the 240 participants, 165 (68.75%) practiced self-medication sometimes and 75 (31.25 %) practiced it often. Self-medication was practiced by 203 (84.58%) respondents for self as well as family members, whereas 37 (15.42%) practiced it for self only.

**Figure 3:**
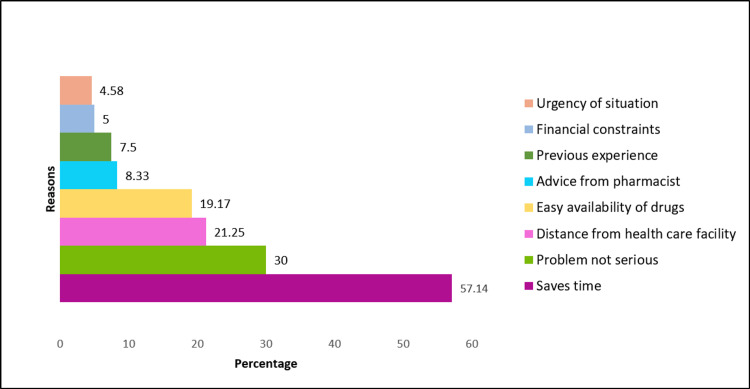
Reasons for self-medication

**Figure 4:**
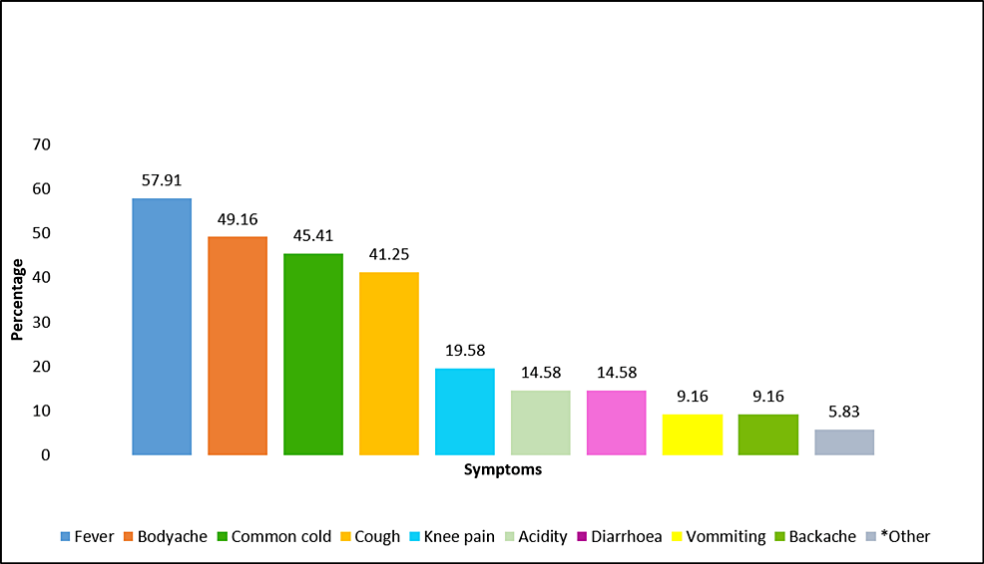
Symptoms for which self-medication was practiced *Other symptoms - dysmenorrhoea, toothache, chest pain

**Figure 5:**
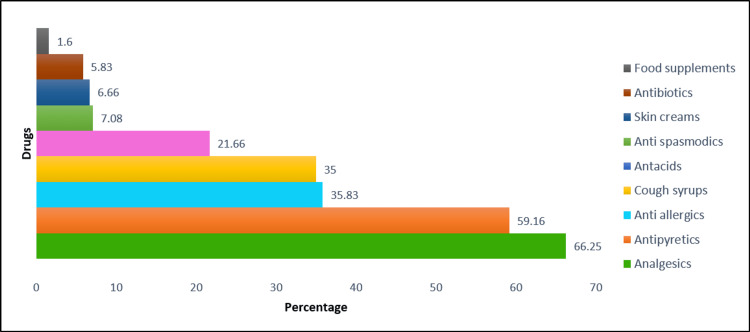
Drugs commonly used for self-medication

The reasons, symptoms, and drugs used for self-medication are seen in Figures 3-5. The commonest reasons for resorting to self-medication as per the findings of the present study are that it saves time (136; 57.14%) and the condition was not serious enough to warrant a physician’s consultation (72; 30%) (Figure 3). Common symptoms for which self-medication was used frequently were fever (139; 57.91), body ache (118; 49.16), common cold (109; 45.41%), and cough (99; 41.25%) (Figure 4). The most widely used drugs for self-medication were analgesics (59; 66.25%) and antipyretics (142; 59.16%). Self-medication was most commonly practiced with allopathic medicines (223; 92.92%), followed by ayurvedic formulations (43; 17.92%) and mixed pathies (22; 9.17%) (Figure 5).

## Discussion

It has become widely accepted that self-medication has an essential place in the healthcare system. Recognition of the responsibility of individuals for their own health and awareness that professional care for minor ailments is often unnecessary to have contributed to this view [7]. Although OTC drugs are meant for self-medication and are of proven efficacy and safety, their improper use due to a lack of knowledge of their side effects and interactions could lead to serious implications, especially in extremes of ages and special physiological conditions like pregnancy and lactation [14].

Prevalence of self-medication

The prevalence of self-medication in the present study was found to be 60%. A similarly high prevalence was also reported by studies carried out by Shamsudeen S et al. [15] and Garofalo L et al. [16]. However, a lower prevalence was reported by Hajira I et al. [17], Jawarkar A et al. [18], and Chari H et al. [19]. The high prevalence observed in the present study can be attributed to the fact that study participants being adults are mainly from the working class who are preoccupied with added responsibilities to be economically stable. This forces them to opt for an easy way out to treat illnesses in the family in ways that save both time and money. The variation in the prevalence observed within and between the countries may be due to the use of different definitions of self-medication, differences in the health-seeking behavior of people, sociocultural factors, and the seasonal variation of diseases.

Factors related to self-medication

In the present study, the relationship between age and self-medication was not found to be statistically significant. In contrast, Kumar V et al. [20] and Alghanim S et al. [21] observed a higher preponderance in younger age groups. The occupation of the study subjects was a significant factor influencing the practice of self-medication in various studies, including the present study. The present study revealed that the higher the level of education, the more the chances of self-medicating. This is in line with the findings of Machado-Alba JE et al. [22], Garofalo L et al. [2], and Awad A et al. [23]. Thus it is evident that the education curriculum is missing out on passing on the message that self-medication involves risk. The results of the present study indicated that pharmacists and family members were the main sources from whom the respondents got information about the choice of drug for practicing self-medication. This finding resonates with that of the study done by Jawarkar A et al. [18] and Sarahroodi S et al. [24]. The reason behind this could be that people tend to follow the advice of their near and dear ones in matters related to health and disease.

Self-medication and symptoms

The most common symptom for which self-medication was practiced in the present study was fever, which is in coherence with the findings of Limaye D et al. [2], Hajira I et al. [17], and Esuvat-Moses A et al. [25]. Commonly consumed drugs by people for the purpose of self-medication in the present study were analgesics and antipyretics. A similar class of drugs was reported to be used for self-medication in studies done by Selvaraj K et al. [26] and Panda A et al. [27].

Reasons for self-medication

The common reasons for self-medication in a study done by Mensur Shafie et al. [28] were perceptions of the mildness of illness, previous knowledge about the medication, and emergency situations warranting self-medication. Familiarity with the treatment or medication was the most commonly cited reason in the study done by Sridhar S et al. [29]. The chief reason for self-medication reported in the present study was that it is “time-saving,” which is similar to the result of Keshari S et al. [30]. Visits to healthcare personnel are often time-consuming and add to the financial burden of a family, making self-medication a more viable option.

Strengths and limitations

The present study is community-based with a sound study design based on considerations such as a prevalence-based sample size, which was substantially large and conducted on a randomly selected representative sample. Owing to the similarities in socioeconomics, health-seeking behaviors, and pharmacy sales practices throughout India, our findings are generalizable to other Indian urban areas.

The cross-sectional nature of the present study precludes inferences about causality.

The present study used a one-year recall period, which might have led to a recall bias. Efforts were made to minimize this bias by using a well-designed, simple, and easy-to-understand questionnaire.

Recommendations

Health education and interventions aimed at changing the perceptions of individuals regarding the accessibility, and affordability of healthcare facility is likely to influence the self-medication practice. Analgesics have harmful effects on the liver and kidneys, so people should be made aware through health education about the adverse effects of its excess use. People should be warned about antibiotic resistance and serious adverse drug reactions of medicines using mass media.

As the pharmacist is the main source of information and provider, they should be made aware of the medicolegal aspects of providing drugs without a prescription. Drug control enforcement needs to be intensified and strict control of sales is highly required.

As most youngsters follow the internet and other mass media advertisements, advertising and selling drugs without a prescription should be banned.

## Conclusions

The prevalence of self-medication was found to be quite high in the present study. It was observed that the female gender and being unemployed or being a homemaker were associated with an increased likelihood of consuming medicines without a prescription from a medical practitioner. Also, it was noted that higher education did not avert people from the harmful habit of self-medicating rather it contributed as an important factor for it. Of those who practiced self-medication, the majority procured the drugs from pharmacists. Hence, it is evident that there is a need to strictly monitor, supervise, inspect, and carry out regular audits to curb the over-the-counter sale of drugs.

Subjects of the present study have self-medicated for minor indications such as fever and body aches. However, self-medicating even for such minor illnesses can prove hazardous and may sometimes lead to fatal complications. Self-medication being a complex phenomenon requires further in-depth research using different study designs and study populations. Findings will help regulators, planners, and health professionals understand the targets of future interventions and develop an integrated strategy to control the practice of self-medication.
